# Heat Shock Factor 1 Mediates Latent HIV Reactivation

**DOI:** 10.1038/srep26294

**Published:** 2016-05-18

**Authors:** Xiao-Yan Pan, Wei Zhao, Xiao-Yun Zeng, Jian Lin, Min-Min Li, Xin-Tian Shen, Shu-Wen Liu

**Affiliations:** 1 Guangdong Provincial Key Laboratory of Drug Screening, School of Pharmaceutical Sciences, Southern Medical University, Guangzhou 510515, China; 2 State Key Laboratory of Organ Failure Research, Guangdong Provincial Institute of Nephrology, Southern Medical University, Guangzhou, China; 3 Center for Clinical Laboratory, First Affiliated Hospital of Jinan University, Guangzhou 510630, China

## Abstract

HSF1, a conserved heat shock factor, has emerged as a key regulator of mammalian transcription in response to cellular metabolic status and stress. To our knowledge, it is not known whether HSF1 regulates viral transcription, particularly HIV-1 and its latent form. Here we reveal that HSF1 extensively participates in HIV transcription and is critical for HIV latent reactivation. Mode of action studies demonstrated that HSF1 binds to the HIV 5′-LTR to reactivate viral transcription and recruits a family of closely related multi-subunit complexes, including p300 and p-TEFb. And HSF1 recruits p300 for self-acetylation is also a committed step. The knockout of HSF1 impaired HIV transcription, whereas the conditional over-expression of HSF1 improved that. These findings demonstrate that HSF1 positively regulates the transcription of latent HIV, suggesting that it might be an important target for different therapeutic strategies aimed at a cure for HIV/AIDS.

The long-lived latent viral reservoir of HIV-1 prevents its eradication and the development of a cure^1^. The recent combination antiretroviral therapy (cART) aimed at inhibiting viral enzymatic activities prevents HIV-1 replication and halts the viral destruction of the host immune system^2^. However, proviruses in the latent reservoir persist in a transcriptionally inactive state, are insuppressible by cART and undetectable by the immune system^3^. Proviruses in the latent reservoir primarily contribute to the rapid rebound of viremia after the cessation of cART^4^. Purging this reservoir is important for the development of a cure for AIDS and requires an understanding of latency mechanisms^5^,^6^,^7^.

In recent years, several host cell proteins and viral regulatory proteins that influence retroviral latency have been identified. The respective lack of cellular transcription factors and HIV-1 Tat protein limits the initiation and elongation of viral transcription in resting CD4^+^ T cells^8^. Genome-wide siRNA screenings have identified a total of 842 host genes associated with HIV-1 infections^9^, and a genome-wide mass spectrometry-based proteomic analysis has revealed 497 HIV-human protein-protein interactions involving 435 individual human proteins in HEK293 and Jurkat cells^10^. Notably, only a small fraction of cellular factors influences the latency maintenance and reactivation stages; thus, it is likely that additional cellular factors exploited by the virus during these stages remain to be identified. These factors might be engaged during transcriptional initiation and subsequent elongation processes.

Until recently, cell stress proteins assisting latent reactivation, except for heat shock protein 90 (HSP90)^11^, have scarcely been detected. Here, we present a previously underappreciated stress protein, heat shock factor 1 (HSF1)^12^, which extensively participates in latent HIV reactivation. First, we identified an important role for HSF1 in latent reactivation under stress. Next, the binding of HSF1 to the HIV long terminal repeat promoter (LTR) was detected during reactivation using chromatin immunoprecipitation (ChIP) assays, and two accessory factors, p300 and positive transcription elongation factor b (p-TEFb), were confirmed to assist HSF1 in maintaining function using co-immunoprecipitation (Co-IP) assays. Moreover, the acetylation of HSF1 was also considered as key point to latent HIV reactivation. Definitively, knockout and overexpression HSF1 further demonstrated its pivotal role in transcription. These data highlight an important role for HSF1 in HIV latency reactivation, providing a deeper understanding of latency mechanisms. These findings suggest that HSF1 might be a novel target for different therapeutic strategies against HIV/AIDS.

## Results

### The cell stress response selectively reactivates HIV in latency cell models

**Figure 1:**
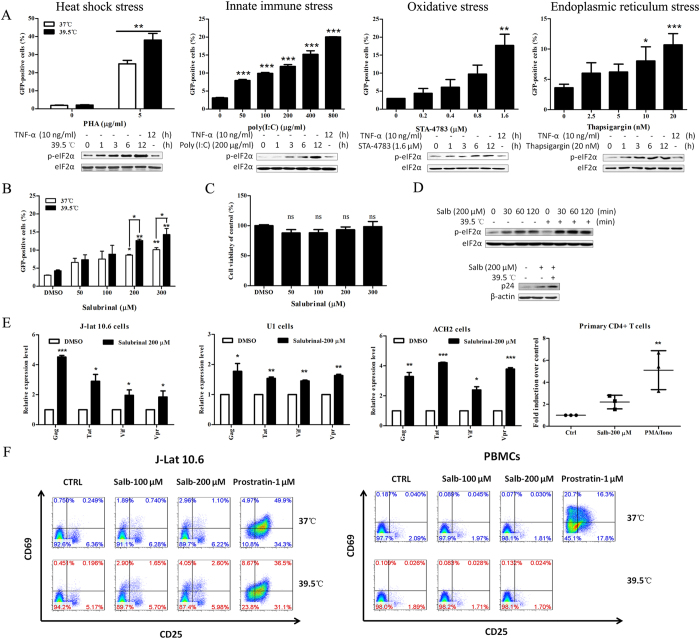
Latent HIV is selectively reactivated by cellular stresses. (**A**) The HIV latency cell line J-Lat 10.6 was stimulated with 5 μg/ml PHA co-stimulated with 39.5 °C, 200 μg/ml poly (I:C), 1.6 μM STA-4783 or 20 nM thapsigargin. GFP expression was examined at 48 hours post-stimulation, and the total levels of p-eIF2α were assayed at 12 hours. (**B**) J-Lat 10.6 cells were treated with salubrinal at the indicated concentrations with or without hyperthermia (39.5 °C) for 48 hours. The percentage of GFP was assessed by FCM. (**C**) The cell viability of J-Lat 10.6 was examined by CCK8 at 48 hours. (**D**) The expression of p24 was detected at 48 hours and p-eIF2α was detected as the figure indicated. (**E**) J-Lat, U1, ACH2 and patient primary CD4^+^ T cells were treated with 200 μM salubrinal or 200 ng/ml PMA plus 2 μM ionomycin for 48 hours. The transcription of HIV genes and 5′-LTR was analyzed by RT-PCR. (**F**) J-Lat 10.6 cells or PBMCs were stimulated with 100–200 μM salubrinal and co-stimulated with 39.5 °C for 24 hours, and 10 μM prostratin was made as control. The expression of T cell activation markers CD69 and CD25 was analyzed by FCM. Data are reported as the mean ± SD from at least three independent experiments. The P value was defined as *p < 0.05, **p < 0.01, and ***p < 0.001 *vs.* control.

To examine latent HIV in response to various cellular stresses, a well established and widely used model of HIV latency based on the Jurkat cell line, J-Lat 10.6^13^ was adopted in the present study. Heat shock, innate immune stress, oxidative stress and endoplasmic reticulum stress are four classical stresses that can be induced by hyperthermia, poly (I:C), STA-4783 and thapsigargin respectively^14^. Stress responses were detected based on the phosphorylation of eukaryotic initiation factor 2 alpha (p-eIF2α) in whole cells. As a result, J-Lat cells treated with these inducers showed significant up-regulation of p-eIF2α expression in 12 hours (Fig. 1A). At the same time, poly (I:C) and STA-4783 increased the expression of GFP over five folds from the basal level, while thapsigargin upregulated the expression of GFP slightly. Hyperthermia (39.5 °C) enhanced PHA-induced GFP expression which was in according with the report by Roesch *et al.*^15^. Although these four inducers influenced latent HIV in different extent, these results indicated that HIV is reactivated from latency in response to cell stress.

We next confirmed the relationship between HIV reactivation and p-eIF2α upregulation after treating cells with a specific eIF2α dephosphorylase inhibitor (salubrinal), which effectively maintains and increases eIF2α phosphorylation. As a result, salubrinal efficiently induced phosphorylation of eIF2α in a short time and this effect was maintained by hyperthermia (Fig. 1D). Consistent with this, salubrinal reactivated latent HIV in a dose-dependent manner and the reactivation was enhanced by hyperthermia (Fig. 1B). The upregulated expression of the HIV capsid protein p24 was consistent with the percentage of GFP (Fig. 1D). Moreover, the salubrinal-mediated reactivation of latent HIV appeared no toxicity to J-Lat 10.6 cells (Fig. 1C). As we observed, salubrinal induced phosphorylation of eIF2α on HIV latency cell model J-Lat 10.6 in our research, which is in agreement with the previous reports showing that it can induce eIF2α phosphorylation in other cell lines^16^,^17^,^18^. The phosphorylation of eIF2α is a marker of stress^19^, while salubrinal can also inhibits ER stress. The possible explanation is that salubrinal-induced phosphorylation of eIF2α triggers HSF1 playing role in cellular proteostasis thus giving cell the ability to resist ER stress in return^20^,^21^.

In addition, ACH2 and U1 cell models, along with primary CD4^+^ T cells from HIV infected individuals receiving suppressive ART were adopted to examine HIV transcription after treatment with salubrinal. Consistently, the transcription of HIV genes such as *gag*, *tat*, *vif* and *vpr* quantitatively analyzed by real-time PCR (RT-PCR) were efficiently upregulated after treatment with salubrinal for 24 hours in the HIV latency cell models ACH2, U1 and J-lat 10.6. Despite that PMA/Iono significantly upregulated the transcription of HIV 5-‘LTR and there was no significance between control group and salubrinal treatment group based on data from three donors, salubrinal could slightly upregulated the transcription of HIV 5′-LTR at 48 hour in primary CD4^+^ T cells from suppressive HIV patients (Fig. 1E).

Interestingly, the latent HIV reactivation stimulated by salubrinal was not associated with global T cell activation. The results on human peripheral blood mononuclear cells (PBMCs) displayed in Fig. 1F showed that the expression of CD25 and CD69 was not up-regulated with salubrinal compared with prostratin treatment. Additionally, the expression of CD25 and CD69 on J-Lat cells was slightly down-regulated following treatment with hyperthermia (39.5 °C). Together, these results indicate that stress sensitizes latent HIV to reactivation across cell models, and this is closely associated with the phosphorylation of eIF2α and the inhibition of global T cell activation.

### HSF1 is activated during HIV reactivation induced by stress inducer salubrinal

**Figure 2:**
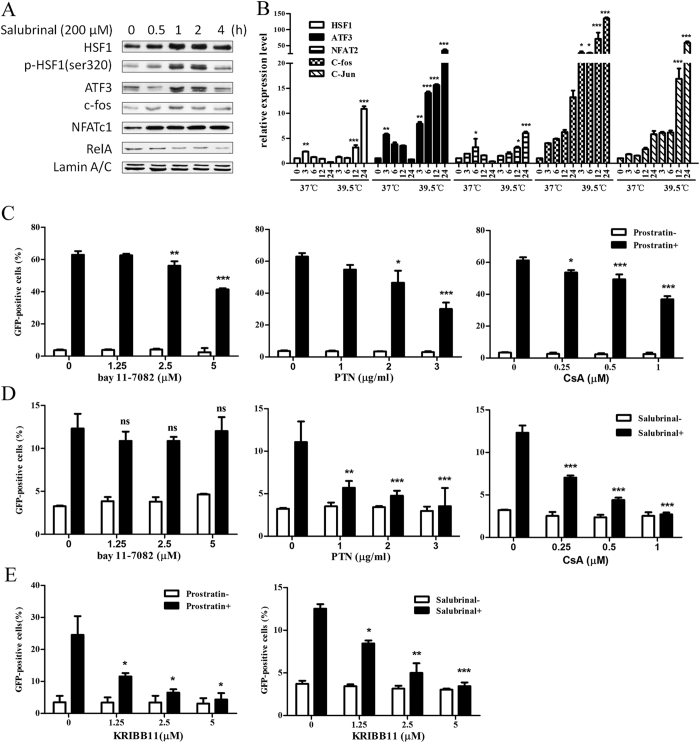
HSF1 is activated during latent HIV reactivation induced by stress. (**A**) The accumulation of cellular transcription factors in nucleus was analyzed after treatment with salubrinal for 4 hours. (**B**) J-Lat 10.6 cells were stimulated with 200 μM salubrinal or co-stimulated with 39.5 °C for 12 hours. The RNA expression of transcription factors were analyzed by RT-PCR. (**C,D**) J-Lat 10.6 cells were treated with 200 μM salubrinal or 10 μM prostratin and co-treated with bay 11–7082, PTN, or CsA as indicated concentrations for 48 hours. The percentage of GFP was examined by FCM. (**E**) J-Lat 10.6 cells were treated with 200 μM salubrinal or 1 μM prostratin and co-treated with KRIBB11. All data are reported as the mean ± SD from at least three independent experiments. The P value was defined as *p < 0.05, **p < 0.01, and ***p < 0.001 *vs.* control (0–37 °C in **B**, 0-salubrinal+/prostratin+ in **C–E**).

We hypothesized that the cellular factors involved in the p-eIF2α downstream signaling pathway might play a major role in reactivation. Therefore, mRNA expression of multiple cellular transcriptional factors (See supplemental Table S1) was examined (Supplemental Figure S1). Indeed, the mRNA expression of AP-1 complex subunits, NFAT and ATF family members and HSF1 were markedly up-regulated. This effect was even more obvious after co-treatment with hyperthermia, and the same tendency was observed in Fig. 2B. As we observed, the gene expression of *c-fos*, *c-Jun* and *ATF3* were significantly upregulated within 24 hour in a time dependent manner under the treatment with salubrinal at 39.5 °C. In comparison, the gene expression of *NFAT2* and *HSF1* changed slightly. Yet, the nuclear import of transcription factors were examined by Western blotting, and phosphorylated HSF1 dramatically accumulated in the nucleus when cells were treated with salubrinal for 2 hours and returned to basal levels after 4 hours (Fig. 2A). The changes in c-fos and ATF3 were consistent with phosphorylated HSF1, and the increased level of NFATc1 remained unchanged in 4 hours. However, RelA which was notably induced by TNF-α, PMA and prostratin^22^, was not up-regulated after salubrinal treatment.

Next, cells treated with salubrinal or the PKC pathway modulator prostratin were respectively co-treated with AP-1 inhibitor (parthenolide, PTN), NFATc1 inhibitor (cyclosporine A, CsA), NF-κB inhibitor (bay 11–7082) or HSF1 –p-TEFb complex inhibitor (KRIBB11). AP-1 and NFATc1 inhibition impaired reactivation whether induced by prostratin or salubrinal, and discrepancy was observed after NF-κB inhibition. The expression of GFP was reduced in J-Lat cells when bay 11–7082 was co-treated with prostratin but persisted when co-treated with salubrinal for 48 hour (Fig. 2C,D). Interestingly, HSF1 inhibition dramatically reduced latent reactivation in a dose-dependent manner, regardless of the reactivation was induced by salubrinal or prostratin (Fig. 2E). These results suggest that multiple transcription factors are involved in latent virus reactivation. As HSF1 and its active form phosphorylated HSF1 (Ser320) showed up a significant increase tendency in the nucleus and GFP% was reduced by KRIBB11 treatments, we deduced that HSF1 may play a role in HIV latency reactivation in addition to the known transcription factors AP-1 and NFATc1. These results also suggest that the latent HIV reactivation induced by salubrinal is HSF1-dependent and NF-κB-independent.

### HSF1 mediates the participation of Heat Shock Proteins (HSPs) in HIV latency reactivation

**Figure 3:**
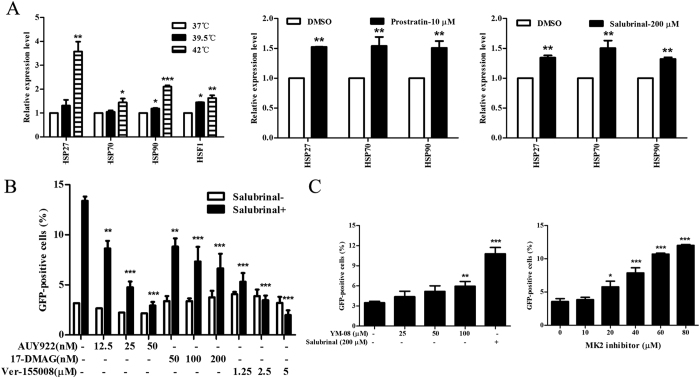
HSF1 mediates HSPs working in latent reactivation. (**A**) J-Lat 10.6 cells were stimulated with 200 μM salubrinal or 10 μM prostratin or treated with hyperthermia (39.5 °C and 40 °C) for 24 hours. Heat shock proteins (HSP90, HSP70, and HSP27) and their transcription factor HSF1were examined using RT-PCR. (**B**) J-Lat 10.6 cells were treated with 200 μM salubrinal or co-treated with HSP90 inhibitor (AUY922 and 17-DMAG) or HSP70 inhibitor (Ver-155008) for 48 hours. The percentage of GFP was examined by FCM. (**C**) J-Lat 10.6 cells were treated with HSP70 activator YM-08 or HSP27 inhibitor MK-2 respectively for 48 hours. The percentage of GFP was examined by FCM. All data are reported as the mean ± SD from at least three independent experiments. The P value was defined as *p < 0.05, **p < 0.01, and ***p < 0.001 *vs.* control (37 °C and DMSO in **A**, 0-salubrinal in **B** and 0 in **C**).

HSF1 is a host transcription factor for HSPs and other stress response genes under stresses^23^, we simply speculated that HSF1 downstream factor may be involved in HIV latency reactivation. The transcription of HSP genes was detected by RT-PCR. As showed in Fig. 3A, the mRNA expression of HSP90, HSP70, HSP27 and HSF1 were up-regulated in J-Lat cells under stimulation with different temperature such as 39.5 °C and 42 °C, and hyperthermia could efficiently enhanced the expression of HSF1 and its downstream genes. Meanwhile, the significant up-regulation of HSP90, HSP70 and HSP27 mRNA expression were observed after treatment with prostratin and salubrinal (Fig. 3A).

To explore whether HSP90, HSP70 and HSP27 influence HIV reactivation, we used AUY922 and 17-DMAG, Ver-155008, and MK-2 inhibitor to inhibit HSP90, HSP70, and HSP27, respectively. Consistent with the results from Anderson *et al.*^11^, HSP90 positively participated in latency reactivation, and the inhibition of HSP90 by AUY922 and 17-DMAG reduced GFP expression in a dose-dependent manner (Fig. 3B). Similar to HSP90, HSP70 inhibition by Ver-155008 also impaired latent HIV reactivation. These two HSPs are positively involved in latent reactivation under the experimental conditions used in the present study. Additionally, the HSP70 activator YM-08 slightly increased GFP% in a dose-dependent manner (Fig. 3C) confirmed the positive role of HSP70 in HIV latency reactivation. In contrast, we presented that HSP27 negatively affected latent reactivation, due to that the inhibition of HSP27 by MK2 inhibitor induced GFP expression rather than reduced that (Fig. 3C). These results implied that HSPs actively express during HIV transcription whether induced by stress inducer salubrinal or non-stress inducer prostratin, and further potentiate our finding that HSF1 plays an important role in latent reactivation.

### HSF1 binds on HIV 5′-LTR and also recruits p-TEFb to take part in transcription regulation

**Figure 4:**
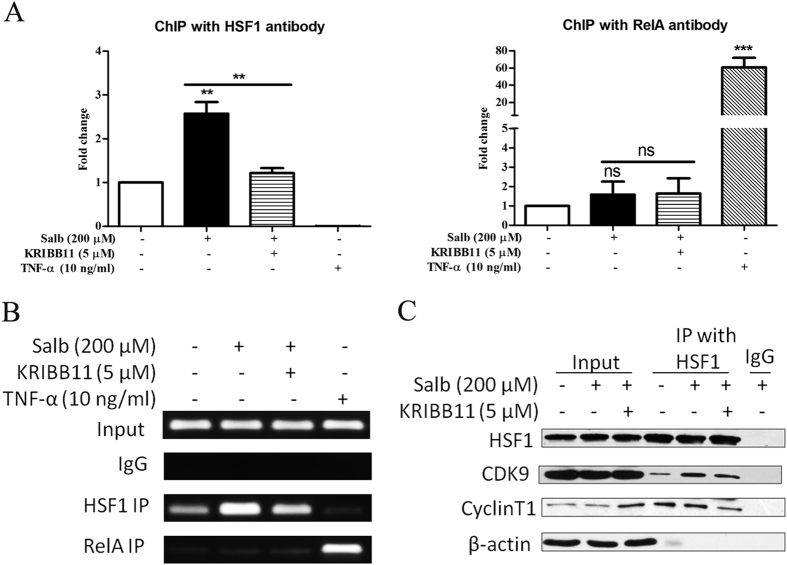
HSF1 binds to HIV 5′-LTR and also recruits p-TEFb reactivate latent HIV in J-Lat cells. (**A**) J-Lat 10.6 cells were stimulated with 200 μM salubrinal or co-treated with KRIBB11 for 2 hours. HSF1 and NF-κB binding on HIV 5′-LTR was detected via ChIP and the relative amount was assessed by RT-PCR. (**B**) The images were from agarose gel electrophoresis. The input was made as 10% total amount and IgG was made as negative control. (**C**) J-Lat 10.6 cells were stimulated with 200 μM salubrinal or co-treated with 5 μM KRIBB11 for 2 hours. The recruitment of p-TEFb complex by HSF1 was detected by Co-IP. IgG was made as negative control. All data are reported as the mean ± SD from at least three independent experiments. The P value was defined as **p < 0.01, and ***p < 0.001 *vs.* control.

To determine how HSF1 contributes to latent HIV reactivation in forward, we examined the impact of HSF1 on HIV 5′-LTR during latency reactivation. According to HSE identified by Rawat *et al.* on HIV 5′-LTR, we designed primers to target on 166 bp fragments (−18 to −183) on HIV LTR containing both HSF1 and NF-κB binding sites. ChIP assays were employed to investigate HSF1 binding on HIV 5′-LTR, in which TNF-α treatment group was made as control. As shown in Fig. 4A, salubrinal increased the amount of HSF1 binding on HIV 5′-LTR over two folds at 2 hour, while TNF-α appeared no influence on that. And the binding of HSF1 to HIV 5′-LTR induced by salubrinal was reduced when co-treated with KRIBB11. In contrast, TNF-α greatly promoted the binding of NF-κB to HIV 5′-LTR while there was hardly none NF-κB binding to HIV 5′-LTR under the stimulation of salubrinal.

With the corresponding to Fig. 4A, the PCR products were conducted agarose electrophoresis and pictures obtained were showed as Fig. 4B. The PCR signal was strong in HSF1 IP group when cells were treated with salubrinal and was weakened with KRIBB11. Meanwhile, there was very weak signal in RelA IP group when cells were treated with salubrinal and was strong with TNF-α. These results further demonstrate that HSF1 binds on HIV 5′-LTR and appears competitive binding with NF-kB.

Next, we investigated the recruitment of cellular transcriptional elongation factors mainly p-TEFb through HSF1. Co-IP experiments have shown that p-TEFb subunit CDK9 and cyclin T1 were recruited by HSF1 in J-Lat cells when treated with salubrinal for 2 hour (Fig. 4C). Meanwhile, the co-treatment with HSF1-p-TEFb complex inhibitor KRIBB11 inhibited the recruitment of p-TEFb to HSF1. As we know, p-TEFb plays a major role in transcriptional elongation by facilitating the continuation of pol II-mediated transcription after the promoter-proximal pause through the CDK9-mediated phosphorylation of the RNA pol II CTD at serine 2^24^,^25^. Moreover, transcriptional elongation is an essential restriction step for latent HIV reactivation. Collectively, the present results demonstrated that HSF1 could bind on HIV 5′-LTR and also recruited p-TEFb, suggesting HSF1′s potential role in HIV transcription as well as transcription elongation.

### HSF1 recruiting p300 for self-acetylation is a committed step for HIV latency reversal

**Figure 5:**
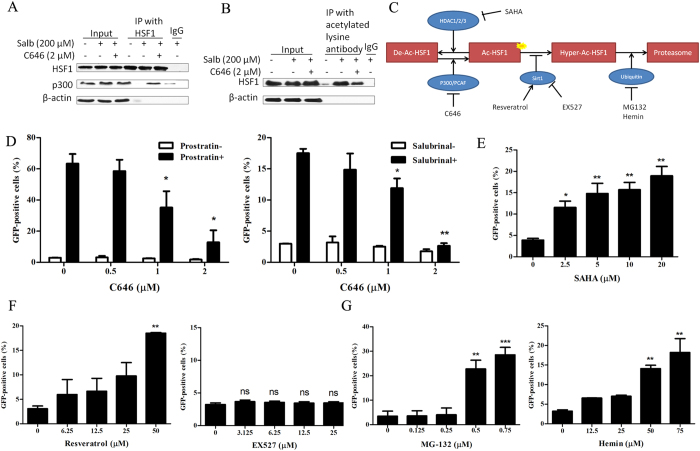
HSF1 recruiting p300 for self-acetylation is a committed step for HIV latency reversal. (**A**) J-Lat 10.6 cells were stimulated with 200 μM salubrinal or co-treated with 2 μM C646 for 2 hours. The recruitment of p300 by HSF1 was detected by Co-IP. IgG was made as negative control. (**B**) The acetylated HSF1 was detected using an HSF1 antibody after IP using pan-acetylated lysine antibody. (**C**) The mode of HSF1 involving with modifiers and their regulators. Acetylase p300 and HDAC played opposite effect on HSF1 acetylation. Deacetylase Sirt1 decreased HSF1 hyper-acetylation. Ubiquitylation happened after HSF1 hyper-acetylation and accelerated HSF1 degradation. (**D**) J-Lat 10.6 cells were treated with 200 μM salubrinal or 10 μM prostratin or co-treated with C646 as indicated for 48 hours. The percentage of GFP was examined by FCM. (**E–G**) J-Lat 10.6 cells were treated with SAHA, resveratrol, EX527, MG-132 and hemin was determined at 48 hours by FCM. All data are reported as the mean ± SD from at least three independent experiments. The P value was defined as *p < 0.05, **p < 0.01, and ***p < 0.001 *vs.* control (0-salubrinal in **D**, 0 in **E–G**).

In addition to p-TEFb, p300 has been detected involving in the complex recruited by HSF1. In Fig. 5A, the binding of p300 on HSF1 was increased under salubrinal treatment at 2 hour, and impaired by p300 inhibitor C646. At the same time, the acetylation of HSF1 was significantly increased in the present of salubrinal and decreased by C646 (Fig. 5B) which was demonstrated by IP experiments with polyclonal acetylated lysine antibody. As a result, the acetylation of HSF1 and the recruitment of acetylase p300 by HSF1 synchronized in the process of HIV reactivation under treatment with salubrinal. Now that the acetylation of HSF1 is happened in the reactivation process, we verified its necessity through inhibiting p300 by C646, and the results is predictable that p300 inhibition impaired latent HIV reactivation induced salubrinal. Similarly, latent HIV reactivation induced salubrinal was inhibited by C646 in a dose-dependent manner (Fig. 5D).

Based on the result that HSF1 recruits p300 to acetylate itself, we speculated that HSF1 acetylation may extensively participates in latency reactivation. On the basis of the report that p300, HDAC, NAD-dependent deacetylase Sirtuin-1 (Sirt1) and proteasome contribute to acetylation of HSF1^26^, we validated p300 inhibitor C646 and HDAC inhibitor SAHA, Sirt1 activator resveratrol and inhibitor EX527, proteasome-ubiquitin inhibitors MG-132 and hemin on J-Lat cells as Fig. 5C indicated. It was observed that SAHA, resveratrol, MG132 and hemin which have been reported to reactivate latent HIV from other pathways, exerted strong ability of latent HIV reactivation in our research, too. SAHA reactivated latent HIV (Fig. 5E) while C646 suppressed reactivation induced by prostratin or salubrinal (Fig. 5D). Resveratrol promoted the activation of latent HIV while EX527 had no effect on that (Fig. 5F). MG132 and hemin both boosted latent HIV reactivation (Fig. 5G). Despite these compounds works on latent HIV reactivation on reported pathways, these results implied that they may also influence latent HIV through acetylizing HSF1. Collectively, we summarized that HSF1 recruiting p300 for self-acetylation is pivotal to the reactivation of latent HIV and the acetylation of HSF1 might extensively participate in latent HIV reactivation, which might provide a better understanding of the mechanisms of HIV-1 latency reversal.

### HSF1 broadly involves in latent HIV reactivation

**Figure 6:**
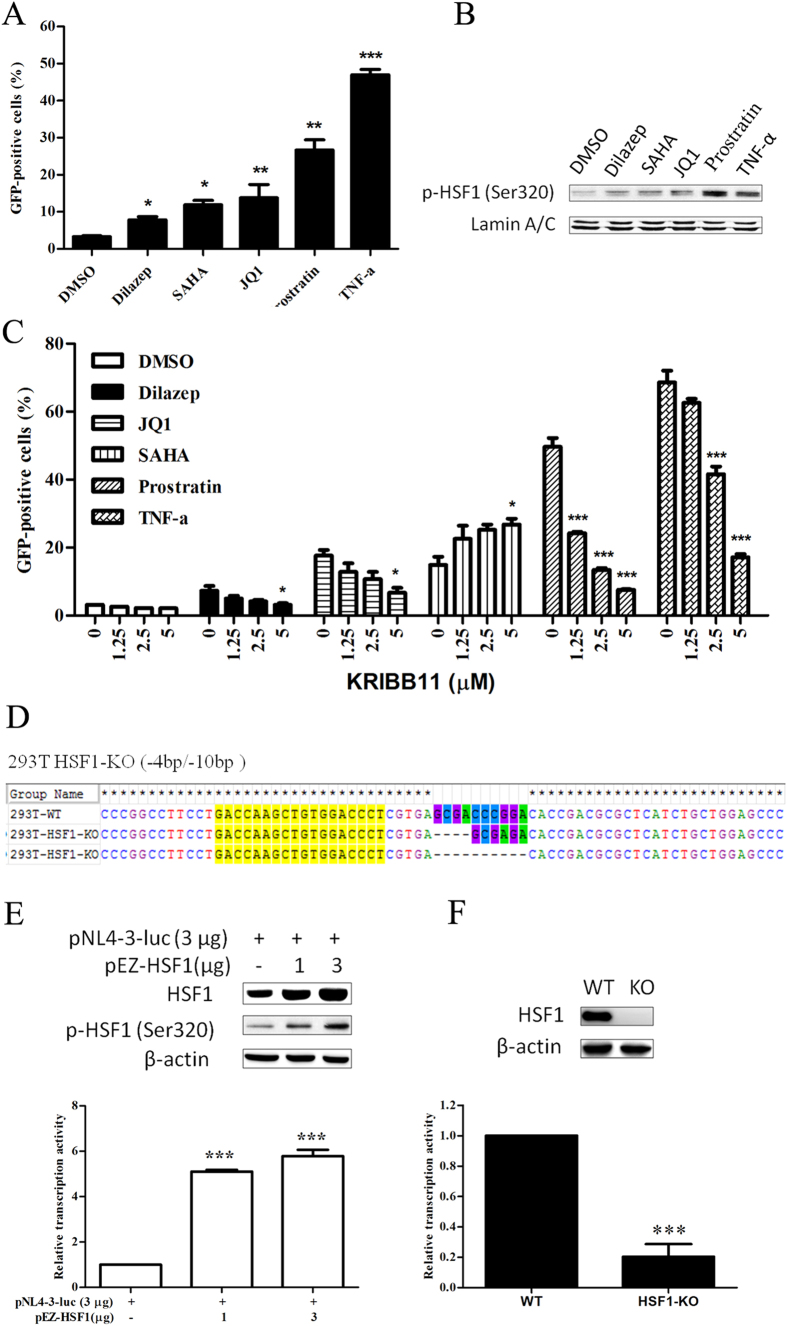
HSF1 plays a critical role in latent HIV reactivation. (**A**) J-Lat 10.6 cells were stimulated with 40 μM dilazep, 1.25 μM JQ1, 1 μM SAHA, 1 μM prostratin, or 10 ng/ml TNF-α for 48 hours. (**B**) The accumulation of p-HSF1 in the nucleus was analyzed at 30 min by Western blotting after treatment with LRAs. (**C**) LRAs as the figure indicated were co-treated with KRIBB11 on J-Lat 10.6 cells, the percentage of GFP was examined by FCM at 48 hours. (**D**) The 293T HSF1-KO (−4/−10 bp) cell line was obtained by TALEN knockout and identified by TA clone sequencing. (**E**) pNL4-3-luci was transfected into 293T-KO cells under normal conditions or co-transfected with pEZ-HSF1 into 293T-WT cells under hyperthermia (39.5 °C) for 24 hours. HSF1 and p-HSF1 was detected by western blot and relative luciferase activity was determined as HIV transcription activity. All data are reported as the mean ± SD from at least three independent experiments. The P value was defined as *p < 0.05, **p < 0.01, and ***p < 0.001 *vs.* control (DMSO in **A**, 0 in **C**, pNL4-3 in **E** and WT in **F**).

To verify whether the requirement of HSF1 for latent reactivation is universal, we co-treated latency reversing agents (LRAs) which reactivate latent HIV through different signaling pathways (Supplemental Table S2) with KRIBB11 on J-Lat cells. As shown in Fig. 6A, HDAC inhibitor SAHA, p-TEFb activators JQ1, PKC agonists prostratin, TNF-α, and dilazep, efficaciously up-regulated GFP%. Meanwhile, the accumulation of phosphorylated HSF1 in the nucleus was markedly increased (Fig. 6B). LRAs co-treated with KRIBB11 were employed to investigate the importance of HSF1 in latency reversal. Except SAHA, and the latency reactivation induced by other four LRAs used in our study were all inhibited by KRIBB11 in a dose-dependent manner (Fig. 6C). As to the reason, one possible explanation was that SAHA might compete with HSF1 for p-TEFb binding, resulting in latent reactivation enhancement. Despite that, we concluded that HSF1 broadly involves in latent HIV reactivation.

In further, we used TALENs to create HSF1 knockout in 293T cells, and we obtained a 293T-HSF1-KO (−4/−10 bp) cell line which was verified by sequencing and then Western blot analysis (Fig. 6D,F). As shown in Fig. 6D, we found 4 bp and 10 bp deletions were generated in HSF1 gene distributing in duplex chromosomes through the analysis of sequences (Fig. 6D). Meanwhile, HSF1 antibody detected no protein signal in 293T-HSF1-KO cells (Fig. 6F). Then HIV plasmid (pNL4-3-luc) was used to transfect 293T-HSF1-KO cells. Relative luciferase activity was detected and normalized to 293T-WT cells. The HSF1 knockout showed significantly reduced luciferase expression and HIV 5′-LTR associated transcription activity (Fig. 6F). The overexpression of HSF1 in 293T-WT cells co-transfected with pNL4-3-luc showed enhanced luciferase activity in a dose-dependent manner under hyperthermia (Fig. 6E), while cells transfected with HSF1 without hyperthermia showed luciferase activity inhibition (Supplemental Figure S2). Phosphorylated HSF1 was augmented along with naked HSF1 under hyperthermia was detected by WB (Fig. 6E). Therefore, HSF1 is an indispensible element in HIV transcription, and activated HSF1 is a major contributor to HIV transcription.

## Discussion

Many cellular factors influence retroviral reactivation. NF-κB^27^, AP-1^28^, NFAT^29^, Ets-1^30^, p-TEFb and p300 directly interact with HIV 5′-LTR, extensively participating in gene transcription. HSF1 is another cellular factor reported in the present study, which largely participates in reactivation in the presence or absence of stresses (Fig. 6). Rawat *et al.* firstly demonstrated that HSF1 is induced during acute HIV-1 infection and positively regulates HIV-1 gene expression through two distinct pathways^31^. Although HSF1 has been reported as a positive regulator of HIV replication^32^, we examined the relationship between the modality and function of this factor in latent HIV reactivation. Indeed, we propose that phosphorylation and acetylation are essential to the positive potency of HSF1, while the preliminary and ubiquitylated forms of this protein manifest negative or no potency, consistent with a previous report^26^.

Mono HSF1 is a repressor of HSP90 and HSP70 in the cytoplasm without any stimulators^33^, and mono HSF1 is transformed into a phosphorylated trimer then promote transcription under stress^34^. It then translocates into the nucleus and binds to HSE^32^, followed by acetylation by p300 for gene activation and ubiquitylation by ubiquitin for HSF1 degradation^35^. This mechanism might explain why the cotransfection of HSF1 with pNL4-3 does not up-regulate the transcription of HIV, suggesting that the active form of HSF1 is necessary. Beyond that, previous study showed that the mutant form of HSF1 (HSF1_ ϪRD) posseses transcriptional activity^36^. However, the precise post-translational modifications controlling HSF1 function remain unclear, and additional studies should focus on the phosphorylation, sumoylation, ubiquitination, and acetylation of HSF1 in novel primary latent models.

Besides that HSF1 is induced by stress, we demonstrate that HSF1 is universal factor in latent HIV reactivation induced by LRAs and indispensable in HIV transcription. Particularly, the p-eIF2α-induced HSF1 activation reversed HIV latency was not associated with global T cell activation. Here we speculate that HSF1 is the crucial factor mediating this process. Besides HSF1 has been reported suppressing NF-κB induced by TNF-α^37^ or heat shock^38^, the binding sites of NF-κB and HSF1 were overlapped in HIV 5′-LTR as show in the previous report^32^. Therefore, phosphorylated HSF1 trimer in nucleus has possibility of occupying NF-κB binding sites, thus the competition binding between HSF1 and NF-κB is taken place on HIV 5′-LTR. The same phenomenon was observed in our study (Fig. 4) where salubrinal promoted much HSF1 binding on HIV 5′-LTR while little NF-κB. Because NF-κB signaling pathway activation accelerates release of immune factors which may facilitate T cell activation and proliferation^39^, the competition of NF-κB and HSF1 for binding HIV 5′-LTR may be one of the reason accounting for latent HIV reactivation by stress without global T cell activation. It needs further study to reveal underlying mechanism.

**Figure 7:**
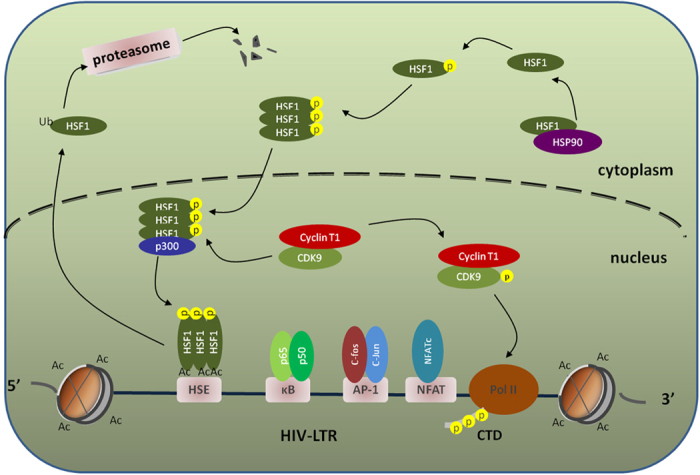
Mechanisms of HSF1 mediate latent HIV-1 reactivation in CD4^+^ T cells. Following stress stimulation or drug treatment, HSF1 is phosphorylated and spontaneously trimerized. Subsequently, HSF1 trimer translocates into nucleus. In nucleus, p300 is recruited to acetylate HSF1, and the acetylated HSF1 trimer binds sites at the HIV-1 LTR. This course is followed by HSF1-dependent elongation, in which acetylated HSF1 recruits the p-TEFb complex to TAR, and CDK9 subsequently phosphorylates the CTD of RNA pol II. Instantly, latent HIV begins transcription initiation and elongation.

In conclusion, the results of the present study showed that HSF1 is an essential cellular factor for regulation of transcription and translation in CD4^+^ T cells, and HIV hijacks this protein to promote viral proliferation. In uninfected cells, HSF1 is conditionally active to facilitate protein refolding and cell survival as a primary molecule regulating the cell stress response^40^. In contrast, HSF1 is constitutively active in latent T cells to participate in latent HIV reactivation. Thus, the breadth of HSF1 biology is far greater than previously appreciated (Fig. 7). The results in present study indicated that latent HIV reactivation mediated by HSF1 may results from many factors such as fever, starvation or special drug stimulation. This may also account for latent HIV stochastically and spontaneously reactivates *in vivo*. Bypass, HSF1 and the associated post-translational modification enzymes identified in the present study provide further information for therapeutic development. Combined with anti-tumor therapy, the promotion of HSF1 activation might be an appropriate solution to antagonize provirus, resulting in the development of a cure for AIDS.

## Methods

### Reagents, plasmids and cell culture

EIF2α, p-eIF2α (Ser51), RelA, NFATc1, c-fos, ATF3, HSF1, p-HSF1 (Ser320), p24, Lamin A/C, β-actin antibodies and secondary antibodies were purchased from Cell Signaling Technology (Beverly, MA, USA). Acetylated lysine antibody, p300, CDK9 and Cyclin T1 antibodies were purchased from Santa Cruz Biotechnology (Santa Cruz, CA, USA). Human FITC conjugated anti-CD25 and PE conjugated anti-CD69 antibodies were purchased from BD Biosciences (San Jose, CA, USA). PHA, poly I:C, STA-4783, thapsigargin, prostratin, PMA, ionomycin, SAHA, JQ1, dilazep, TNF-α, hemin, salubrinal, MG-132, BAY 11-7082, CsA, C646 and EX527 were from Sigma-Aldrich (St. Louis, MO, USA). Resveratrol, parthenolide (PTN), Ver-155008, KRIBB11, 17-DMAG and NVP-AUY922 were from Merck Calbiochem (Darmstadt, Germany). PEZ-HSF1 was purchased from ViGene Bioscience Inc (MD, USA). NL4-3E-R-luc plasmid, J-Lat 10.6^13^, U1^41^ and ACH2^42^ cell lines were obtained from the National Institutes of Health AIDS Research and Reference Reagent Program.

J-Lat 10.6, U1 and ACH2 cell lines were maintained in RPMI1640 (Gibco, Grand Island, NY) supplemented with 10% fetal bovine serum (Gibco) at 37 °C with 5% CO_2_. High temperature treatment (39.5 °C) was operated by putting cells in another incubator which temperature was adjusted to 39.5 °C.

### Primary CD4^+^ T cell isolation and treatment

PBMCs were isolated by density gradient centrifugation using Histopaque-1077 (Sigma-Aldrich), the peripheral blood samples from HIV positive patients and health person were collected in The Eighth People’s Hospital of Guangzhou (Guangzhou, China) and Nanfang Hospital (Guangzhou, China) with informed consents. The experiment was approved by the Ethical Committee of Nanfang Hospital and performed in accordance with relevant guidelines and regulations. The HIV-infected patients were selected based on sustained plasma viral load suppression (plasma viral loads were <20 copies/ml for >12 months and CD4 count >350 cells/μl), and their essential information were listed in Table S3. The primary CD4^+^ T cells were isolated by EasySep kit (STEMCELL Technologies Inc. Vancouver, BC, Canada) according to manufacturer’s instruction from PBMCs and the purity was >90%. 4 × 10^6^ cells were incubated with 200 μΜ salubrinal or 200 ng/ml plus 2 μM ionomycin for 48 h. Then cells were collected to be conducted total RNA extraction and real time-PCR experiment with HIV 5′-*LTR* primers as follow.

### Cell viability assay

Cell viability was evaluated by CCK-8 kit (Dojindo Molecular Technologies, Inc., Japan). J-Lat 10.6 and PBMCs plated as 1 × 10^6^ cells and 2 × 10^6^ per well respectively in 96-well plate were incubated with compounds for 48 h. Then 10 μl of CCK-8 reagent were added to 100 μl of cell culture mixture and incubated for additional 4 h at 37 °C. Optical density (OD) was recorded at wavelength of 450 nm on a micro-plate reader (TECAN, Swiss).

### Flow cytometry (FCM)

After drug treatment, the cells were analyzed by flow cytometry to calculate the percentage of GFP-positive cells using the FITC channel (BD FACS Canto II, San Jose, CA) to determine the level of HIV-1 gene expression. Cell surface staining was performed at 4 °C for 30 min in the dark using CD25-PE- and CD69-APC-conjugated antibodies after drug treatment for 24 h. The cells were washed twice with phosphate buffered saline (PBS) and analyzed through dual channel with fluorescence compensation. The fluorescence values and dot plots were analyzed using FlowJo 7.6 software (Treestar, San Carlos, CA).

### Real-time PCR

J-Lat 10.6, U1, ACH2 and primary CD4^+^ T cells were treated with salubrinal (200 μM) for different times, and total RNA was extracted using TRIZOL (Invitrogen, Carlsbad, CA). Reverse transcription was performed using the PrimeScript RT reagent Kit with gDNA Eraser (TAKARA, Japan). Real-time PCR was performed using SYBR Select Master Mix (TAKARA) on the 7500 Real-Time PCR System (Applied Biosystems, Foster City, CA) using a standard two-step procedure (denature: 95 °C/15 sec, anneal/extend: 60 °C/1 min, 40 cycles). The primers are list in supplemental Table S1. The 2^−ΔΔCT^ method was adopted to analyze relative gene expression levels and *GAPDH* gene was made as reference gene in all experiments.

### Protein extract and Western blot analysis

After treatment, the cells were lysed in RIPA (50 mM Tris-HCl, pH 7.5, 150 mM sodium chloride, 1 mM EDTA, 1% Triton-X-100, 0.25% sodium deoxycholate, 0.1% SDS) containing a 1 × protease and phosphatase inhibitor cocktail (Merck Calbiochem) and incubated on ice for 10 min, followed by centrifugation at 12,000g for 10 min at 4 °C. The supernatant was collected as a whole protein extract. The nucleoprotein was extracted using NE-PER nuclear and cytoplasmic extraction reagents (Thermo Fisher Scientific, Carlsbad, CA) according to the manufacturer’s protocol. Total protein was quantified, and a standard curve was generated. SDS sample buffer was added to each sample, followed by denaturation at 100 °C for 10 min, electrophoresis for 1.5 h on a 10% polyacrylamide gel to separate the proteins, transfer onto PVDF membranes, and co-incubation with primary and secondary antibodies conjugated to HRP. Subsequently, ECL substrate was added, and the blot was exposed to film to develop the image.

### Chromatin Immunoprecipitation (ChIP)

J-Lat 10.6 cells (6 × 10^6^) were treated with salubrinal or co-treated with KRIBB11 for 2 hours, and TNF-α was made as control. Cells were harvested and fixed with 0.75% formaldehyde for 10 min at room temperature, then lysed to obtain nuclear extract and digested to obtain chromatin segments (200–1000 bp) using the ChIP assay kit (Thermo Fisher Scientific) according to manufacturer’s instructions. The input controls were made as 10% nuclear extract. HSF1 and RelA monoclonal antibodies (ChIP grade) were adopted to incubate with nuclear extract respectively at 4 °C overnight, and IgG was made as negative control. Protein A/G was added to collect the immune complex and incubate with shaking for 1 h at 4 °C. DNA were purified and recovered for RT-PCR as the kit instructions. The sequence of primers used for PCR amplification of HIV-LTR is as following: forward (−183) 5′-GCCTCCTAGCATTTCGTCACAT-3′ and reverse (−18) 5′- GCTGCTTATATGTAGCATCTGAGG-3′. The amplified DNA products (166 bp), which contained the cis-acting element for both HSF1 and NF-κB, were subjected to 2% agarose gel electrophoresis and images were captured under the UV transilluminator (Alpha Innotech, CA, USA).

### Co-immunoprecipitation (Co-IP) and immunoprecipitation (IP)

Approximately 5 × 10^6^ J-Lat 10.6 cells were treated with salubrinal or co-treated with KRIBB11 or C646 for 2 h, and protein extracts were obtained after the addition of 300 μl of IP lysis buffer (20 mM Tris, pH 7.5, 150 mM NaCl, 1% Triton X-100) and lysis on ice for 30 min. The samples were centrifuged at 14,000 rpm for 10 min, and 1 μg of mouse or rabbit IgG was added to the supernatant followed by incubation for 30 min at 4 °C. Subsequently, 30 μl of protein A/G plus-agarose (Santa Cruz) was added, and the mixture was incubated for another 30 min at 4 °C during the precleaning step. After centrifugation at 2500 rpm for 5 min to obtain supernatant, 2 μg of antibody (HSF1 or acetyl-lysine) was added to 300 μg of protein and incubated with shaking at 4 °C overnight. The input sample contained 20 μg of protein. The next day, 30 μl of protein A/G plus agarose was added, and the mixture was incubated for another 4 h at 4 °C. The mixture was centrifuged, washed three times with 500 μl IP lysis buffer containing 0.1% PMSF, 1X loading buffer was added to resuspend the precipitation, and the sample was then boiled at 100 °C for 10 min. Finally, the samples were analyzed by Western blot.

### HSF1 TALENs knockout

TALENs arms were designed as L2*R3 combination targets on the HSF1 gene (NCBI gene ID: 3297), and the plasmids for the TALENs left and right arms were constructed using the FAST TALEN Kit (SIDANSAI, China). After sequencing, five plasmids were loaded into 293T cells in a L2*R3 cross combination. One pair of arms was selected as the most effective knockout group after 3 days of puromycin screening and subsequent genomic PCR sequencing. Another round of screening was performed after a two-week monoclonal culture. The stable knockout cell line was acquired and identified by sequencing and Western blotting.

### Plasmid transfection and luciferase activity assays

293T cells in the exponential growth period were split at 5 × 10^5^ cells per well and seeded into a 6-well plate. PNL4-3-luc, pEZ-HSF1 and its associated control plasmid (pEZ-NG) were mixed with PEI (polyethylenimine), followed by co-incubation with the cells for 24 h. The cells were lysed, and the relative luciferase activity was detected using a Dual-Luciferase Reporter Assay System (Promega) according to instruction. The substrate and luminescence were scanned using a microplate reader with a white plate. Relative transcription activity was normalized according to renilla luciferase activity then compared with pNL4-3-luc and pEZ-NG co-transfection group.

### Statistical analysis

All the experiments described were independently repeated at least three times to verify reproducibility. The data were analyzed using Prism 5.0 software, and the error bars are presented as the means ± SD from at least three independent experiments. The statistical analysis of the experimental data was performed using one-way ANOVA with Dunnett’s test, and the P value defined as *p < 0.05, **p < 0.01, and ***p < 0.001.

## Additional Information

**How to cite this article**: Pan, X.-Y. *et al.* Heat Shock Factor 1 Mediates Latent HIV Reactivation. *Sci. Rep.* **6**, 26294; doi: 10.1038/srep26294 (2016).

## Supplementary Material

Supplementary Information
