# Supplementary material for: Heat Shock Factor 1 Mediates Latent HIV Reactivation
Source: Sci Rep. 2016 May 18;6:26294. doi: 10.1038/srep26294 (PMC4870680; doi:10.1038/srep26294)
Supplement: Supplementary Information [file srep26294-s1.doc]

**Heat Shock Factor 1 Mediates Latent HIV Reactivation**

**Xiao-Yan Pan^1,#^, Wei Zhao^1,2,#^, Xiao-Yun Zeng^1^, Jian Lin^1^, Min-Min Li^3^, Xin-Tian Shen^1^, Shu-Wen Liu^1,2,*^**

^1^Guangdong Provincial Key Laboratory of Drug Screening, School of Pharmaceutical Sciences, Southern Medical University, Guangzhou 510515, China; ^2^State Key Laboratory of Organ Failure Research, Guangdong Provincial Institute of Nephrology, Southern Medical University, Guangzhou, China; ^3^Center for Clinical Laboratory, First Affiliated Hospital of Jinan University, Guangzhou 510630, China.

*Correspondence to: S. Liu, Tel: +86-20-61648538, Fax: +86-20-61648655, E-mail: liusw@smu.edu.cn

^#^ Xiaoyan Pan and Wei Zhao contributed equally to this paper.

## Supplementary Information

**Figure Legends**

**Figure S1.** **The mRNA expression of cellular transcriptional factors during salubrinal treatment.** J-Lat 10.6 cells were stimulated with 200 μM salubrinal or co-treated with hyperthermia (39.5°C) for 12 hours. The mRNA expression of cellular transcriptional factors was examined by RT-PCR.

**Figure S2. HSF1 Overexpression.** PEZ-HSF1 was co-transfected with pNL4-3-luc into 293T cells, and relative luciferase activity was detected at 24 hours after transfection. HSF1 overexpression was validated by Western blotting.

**Figure S3. Cell viability of J-Lat 10.6 cell treated with inhibitors.** J-Lat 10.6 cells were co-treated with inhibitors such as bay 11-7082 (1.25, 2.5, 5 μM), PTN (1, 2, 3 μg/ml), CsA (2.5, 5, 10 μM), KRIBB11 (1.25, 2.5, 5 μM) with C646 (0.5, 1, 2 μM) and salubrinal (200 μM) or prostratin (1 μM) respectively for 48 h. Cell viability was detected by CCK8, and calculated as OD_sample_×100%/ OD_DMSO_.

**Figure S4. Gray quantitative analysis for western blotting bands related to Figure 1.**

**Figure S5. Gray quantitative analysis for western blotting bands related to Figure 2.**

**Figure S6. Gray quantitive analysis for western blotting bands related to Figure 4 and Figure 5.**

**Figure S7. Gray quantitative analysis for western blotting bands related to Figure 6 and Figure S2.**

**Figures**

**Figure S1.**


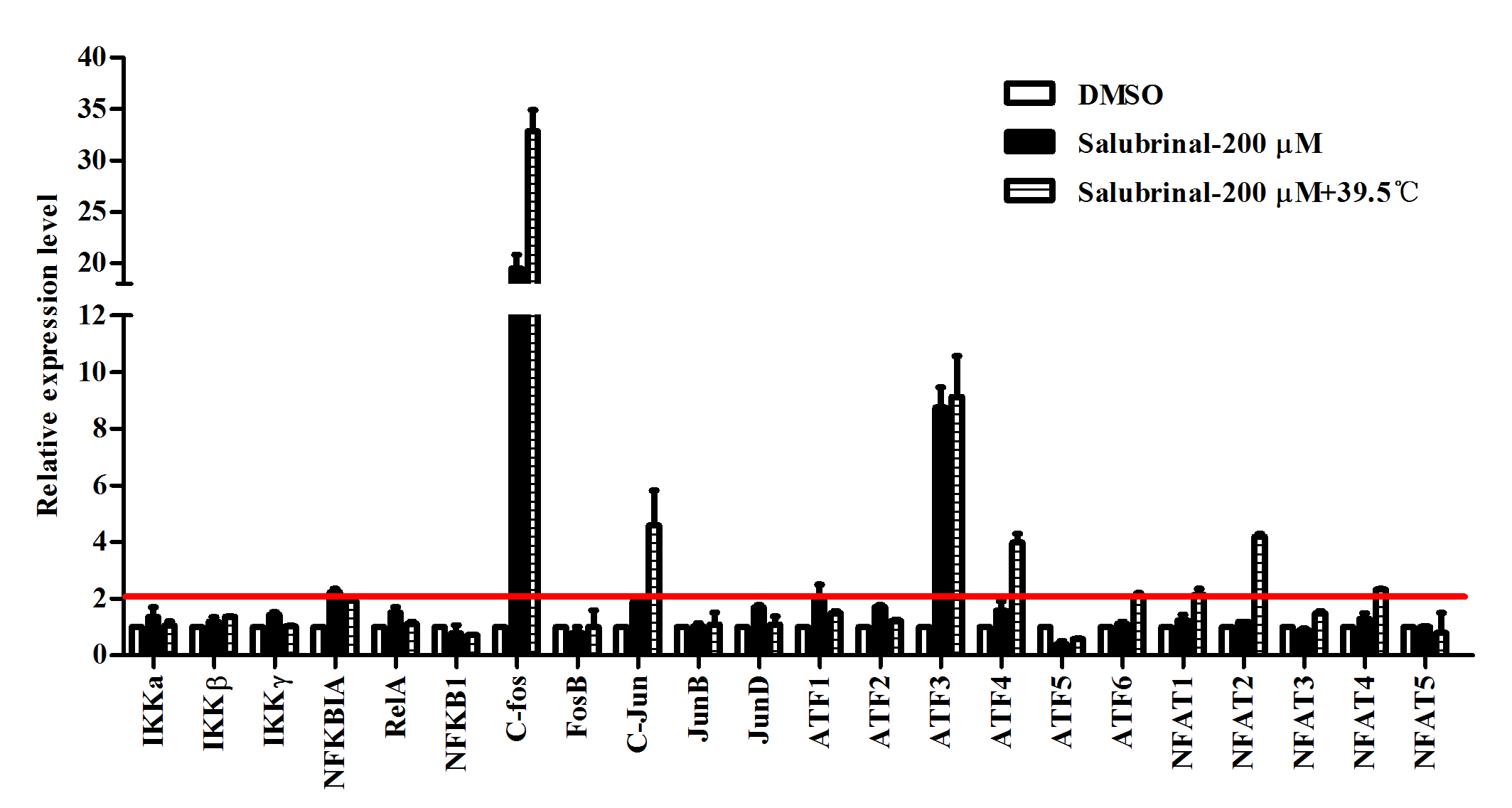


**Figure S2.**

**
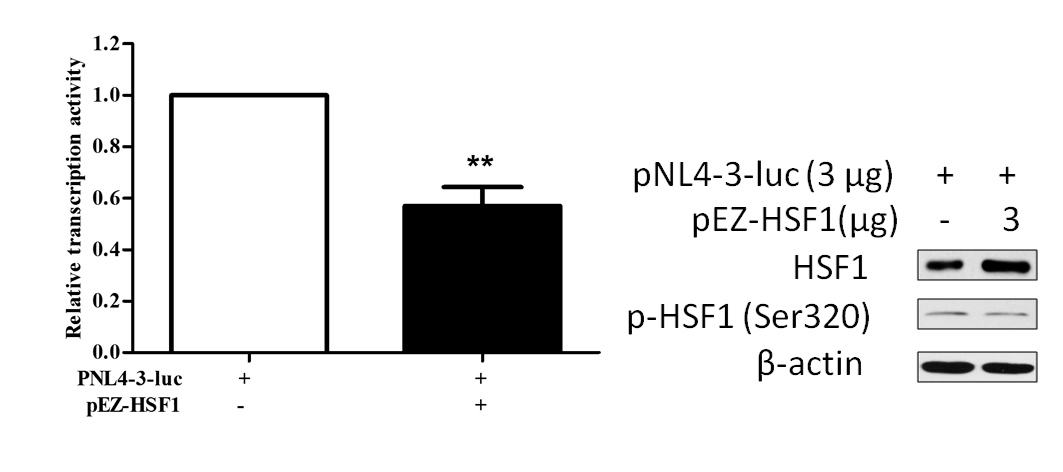
**

**Figure S3.**

**
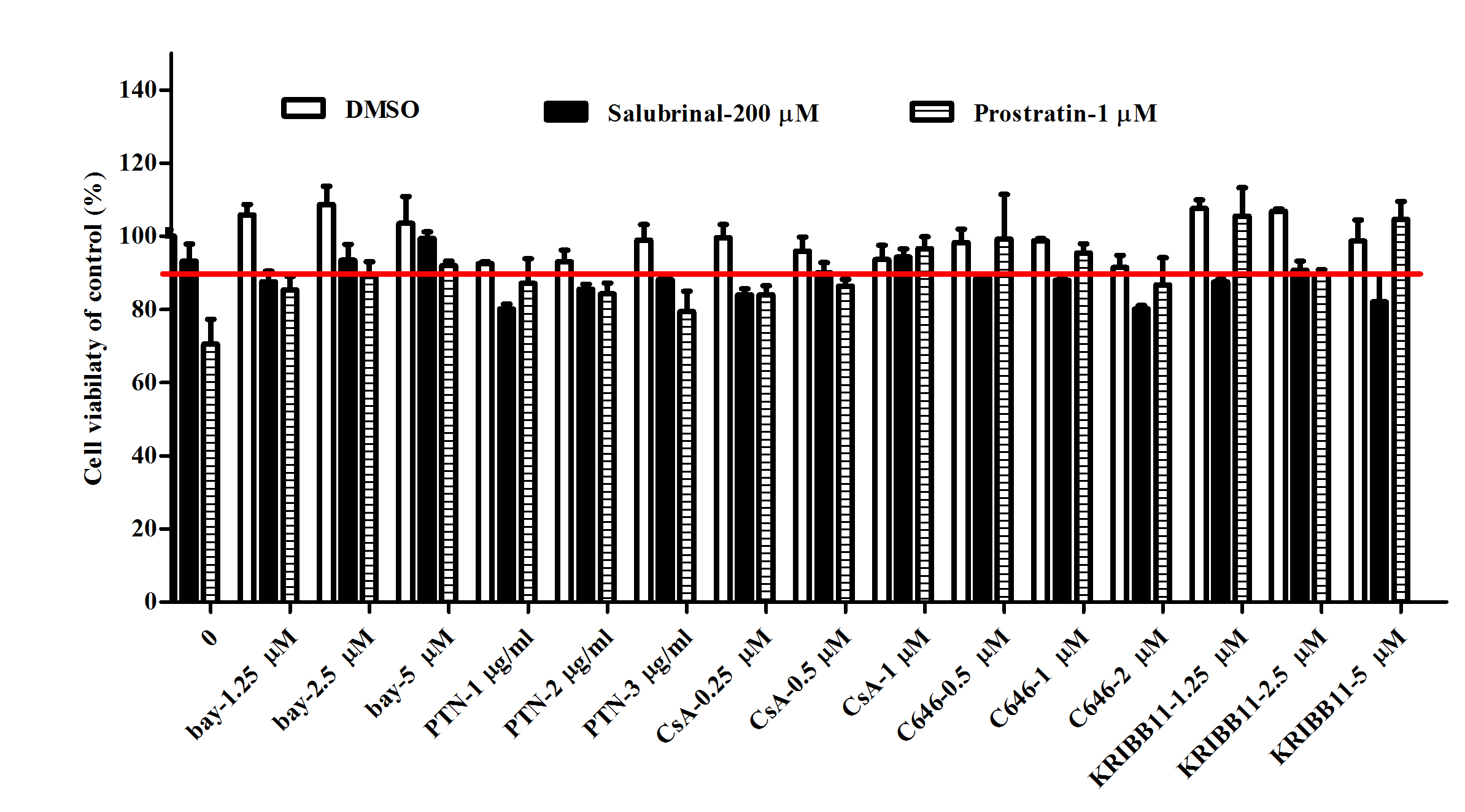
**

**Figure S4.**

**
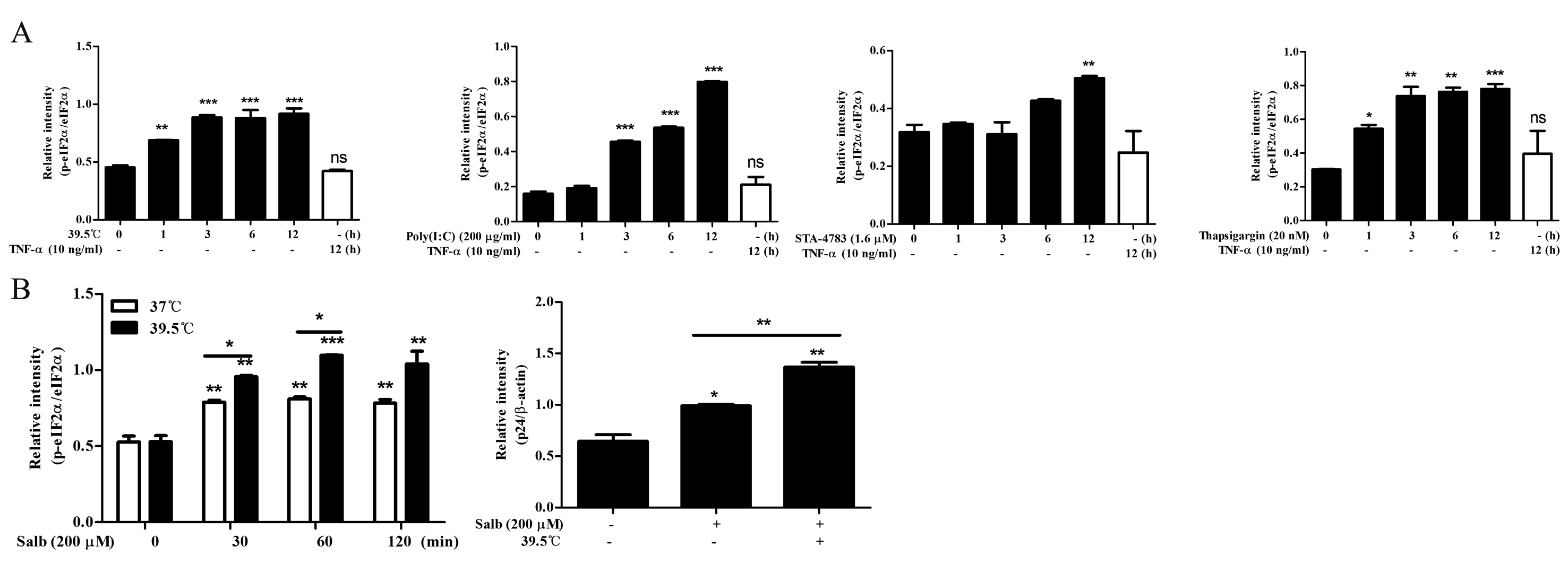
**

**Figure S5.**

**
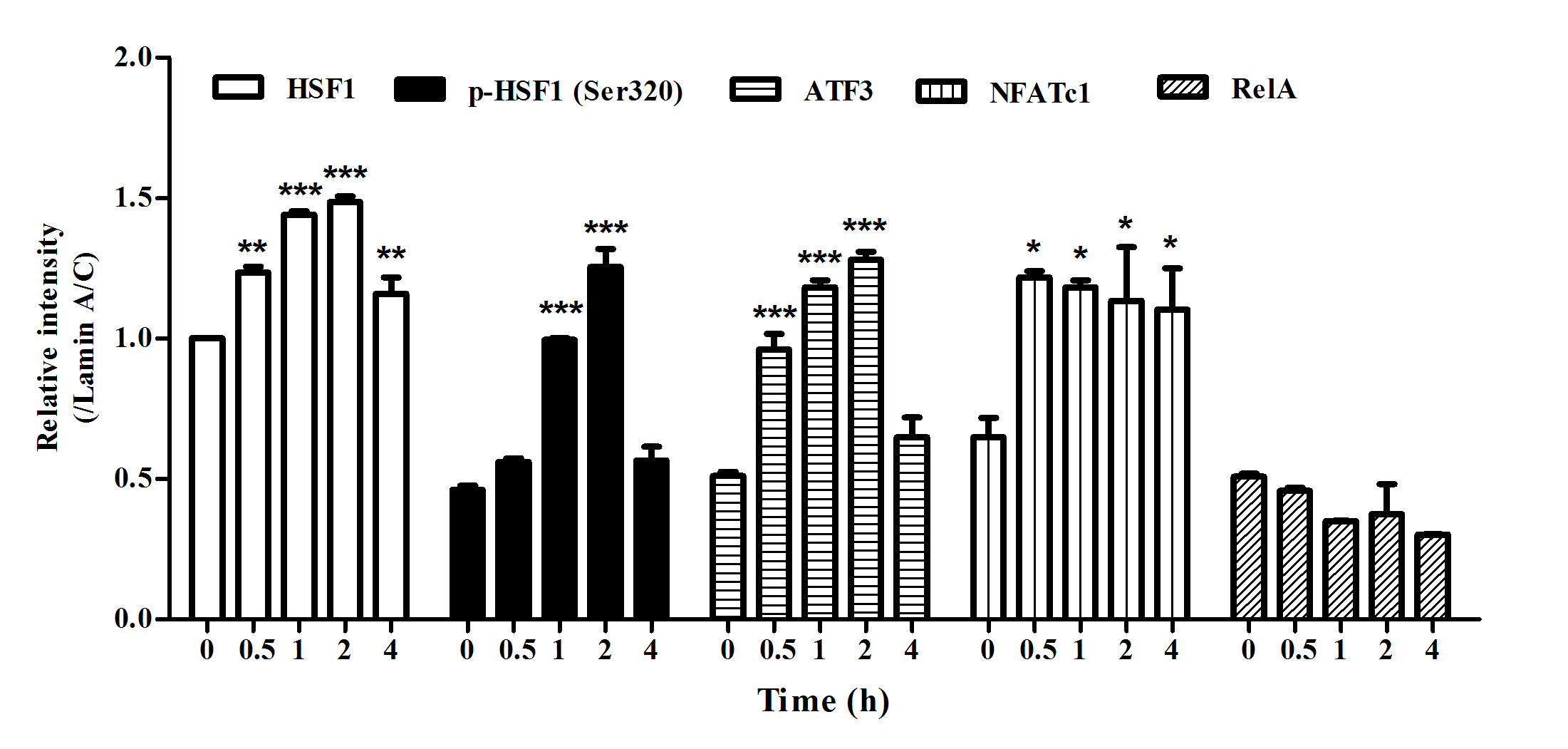
**

**Figure S6.**

**
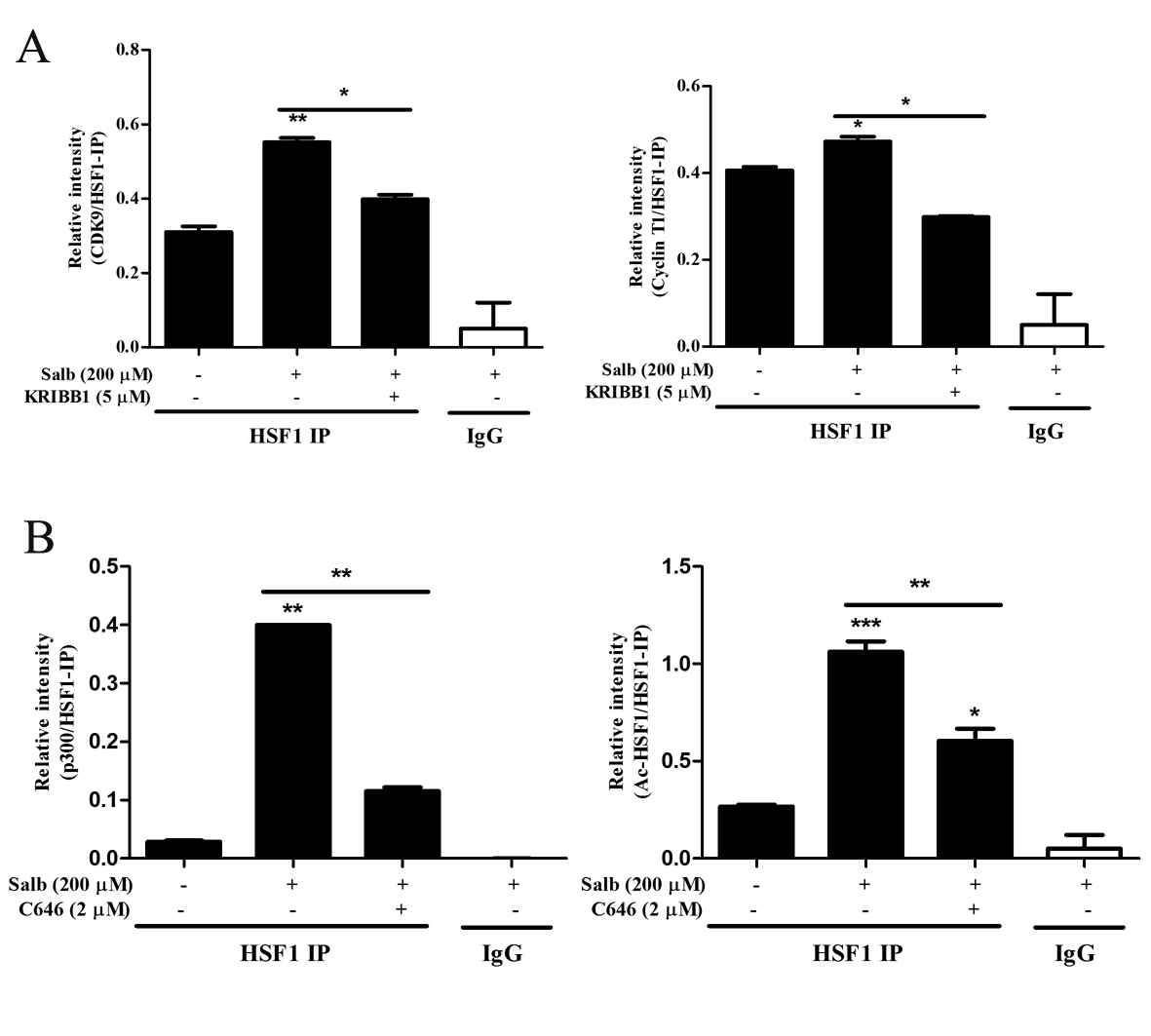
**

**Figure S7.**

**
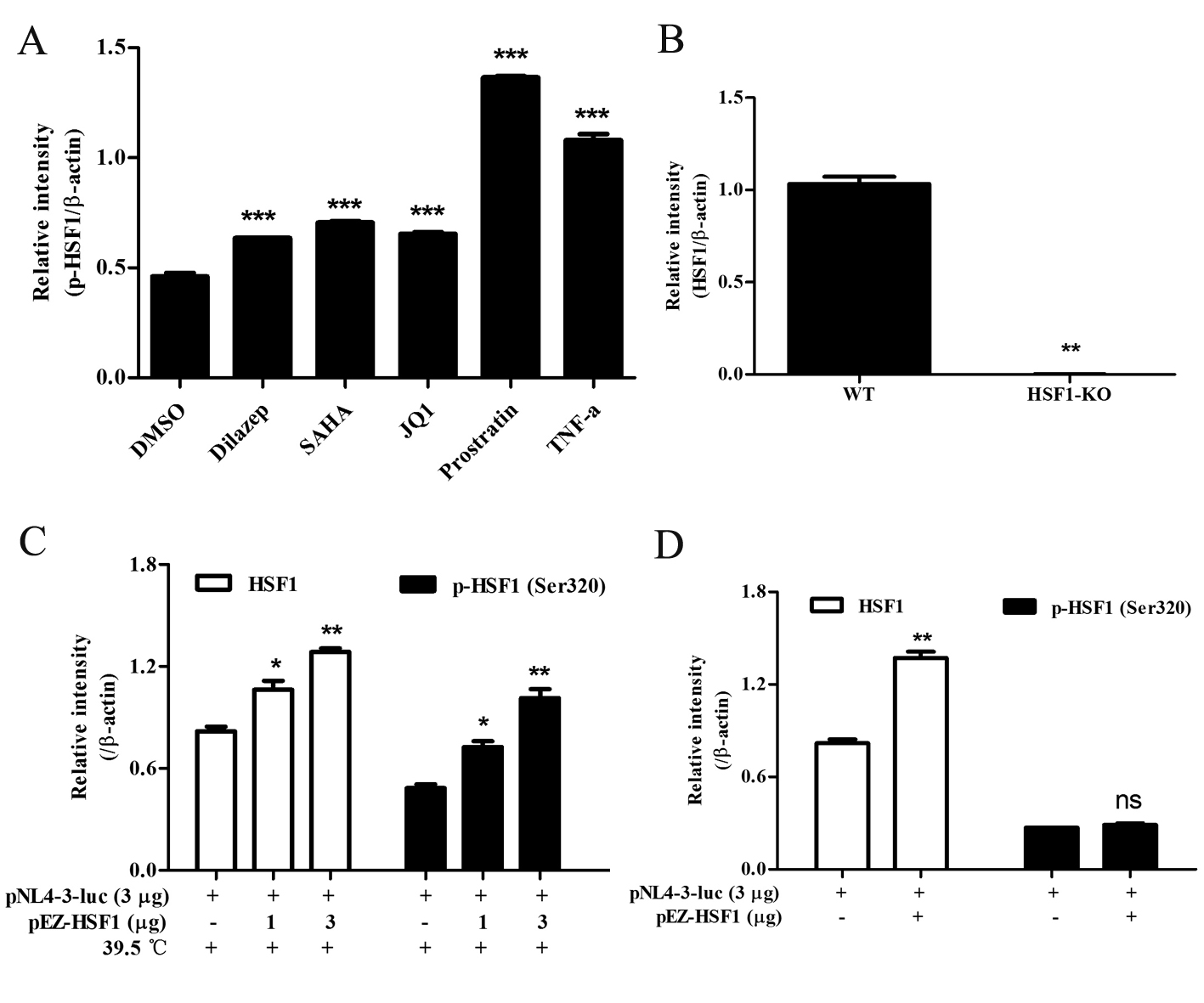
**

**Tables**

**Table S1. RT-PCR primers used in this study.**

| **Primers** | **Sequence** |
| --- | --- |
| ***GAPDH* F**  ***GAPDH* R**  **HSF1 F**  **HSF1 R**  **IKKα (*IKK1,CHUK*) F**  **IKKα (*IKK1,CHUK*) R**  **IKKβ(*IKK2,IKBKB*) F**  **IKKβ(*IKK2,IKBKB*) R**  **IKKγ(*NEMO,IKBKG*) F** | CTCTGCTCCTCCTGTTCGAC  AGTTAAAAGCAGCCCTGGTGA  TCCTGCGGGAGAGTGAACCT  GCTCATTCTTGTCCAGGCAGG  CCCCGACTTCAGCAGAACAT  GCCCTGTTCCTCATTTGCCT  CCCTGGTAAATGACAGTGGGA  TGACTGCTTTGGTTGGCATT  GGCACTGGGGAAGTCAAGAA |
| **IKKγ(*NEMO,IKBKG*) R**  ***NFKBIA* (*IkBA*) F**  ***NFKBIA* (*IkBA*) R**  **RelA (NF-κB p65) F**  **RelA (NF-κB p65) R**  ***NFKB1*(NF-κB p50) F**  ***NFKB1*(NF-κB p50) R**  **c-fos F**  **c-fos R**  **FosB F**  **FosB R**  **c-Jun F**  **c-Jun R**  **JunB F**  **JunB R**  **JunD F**  **JunD R**  **NFATc2 (*NFAT1*) F**  **NFATc2 (*NFAT1*) R**  **NFATc1 (*NFAT2*) F**  **NFATc1 (*NFAT2*) R**  **NFATc4 (*NFAT3*) F**  **NFATc4 (*NFAT3*) R**  **NFATc3 (*NFAT4*) F**  **NFATc3 (*NFAT4*) R**  **NFAT5 F**  **NFAT5 R**  **ATF1 F**  **ATF1 R**  **ATF2 F**  **ATF2 R**  **ATF3 F**  **ATF3 R**  **ATF4 F**  **ATF4 R**  **ATF5 F**  **ATF5 R**  **ATF6 F**  **ATF6-R**  ***HSPB1* (HSP27) F**  ***HSPB1* (HSP27) R**  ***HSPA14* (HSP70) F**  ***HSPA14* (HSP70) R**  ***HSP90AB1*(HSP90) F**  ***HSP90AB1*(HSP90)R**  **Gag F**  **Gag R**  **Tat F**  **Tat R**  **Vif F**  **Vif R**  **Vpr F**  **Vpr R**  **LTR F**  **LTR R** | GCACCCGTGTGCATGGT  GGGCCAGCTGACACTAGAAA  CTCATAACGTCAGACGCTGG  GGGGTTTGGTCTGGGACTTC  AGAGCAAGAGTCCAAGTGCTTT  CAGCAGATGGCCCATACCTT  TTGCAGATTTTGACCTGAGGGT  TCATGCATTGTTGAGGTGGTC  CACATGTCAAAAGACCTCAAGGTAG  AGCAGCAGCTAAATGCAGGA  CCACCAGCACAAACTCCAGA  GCCGGCCTGGCCTTTC  GACAGGGGCGGTGTCTC  CCCTACCGGAGTCTCAAAGC  TGCTGTTGGGGACAATCAGG  CCCCTTTCCTCGATCTCGC  GCTGGCGTAACGAGACTTTA  AGTCCAGTGGCAGAATCGTC  CTGCCCCGTGAGGATCATTT  TACGTCTGCAACGGGAAGAG  CTTTACGGCGACGTCGTTTC  CTTGCCTCACCTTCAGGCTT  AGCAACAGGCGAGGAGTTAC  AATATGTCACAGTTTGTGTGATCC  TCCCAATTATCTCGTTCACATCAT  TCATCTCAGCAGACATCAGGAA  AGTGTTGTCACAGGTGGCTG  CAGGCACAGATGGAGTACAGG  CTGATGCAGTTTGTACGACCA  TCAACCTCCAAGGCAGAAGC  AAACTGTTGTACTGCAGGTTTTTA  TCCTTTCCTTGTGGTTGAAAAC  AAACACCCTGGGCCAGATTT  CCACTAGGTACCGCCAGAAG  GCCTTGCGGACCTCTTCTAT  GAGACCAGAACAAGTCGGCG  CCTTGACGTACTGGATCTCGC  CATGAAGTAGAAAGGACCAAGTCA  TCACTCCCTGAGTTCCTGATAC  CACGCGGAAATACACGCTG  GACTCGAAGGTGACTGGGATG  CGAGTTACTCCAGCTGTTGTTG  CTGAGCTTGTGGATCACTGG  ATATAGGGCGACTTGGGGCAC  GTGCACTTCCTCAGGCATCTAA  GTCCAGAATGCGAACCCAGA  GTTACGTGCTGGCTCATTGC  ATGGAGCCAGTAGATCCTAGACT  CGCTTCTTCCTGCCATAGGA  CACACAAGTAGACCCTGACCT  CCCTACCTTGTTATGTCCTGCT  CCACAAAGGGAGCCATACAATG  TTATGGCTTCCACTCCTGCC  GCCTCCTAGCATTTCGTCACAT  GCTGCTTATATGTAGCATCTGAGG |

**Table S2. Latent activators used in this study.**

| **Reagents** | **Structure** | **Signaling pathways**  **/target molecular** |
| --- | --- | --- |
| **Prostratin** | 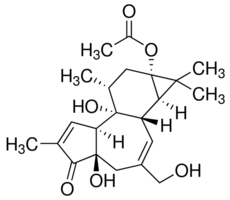 | PKC/NF-κB |
| **SAHA**  **TNF-α**  **JQ1**  **Dilazep**  **Salubrinal**  **Resveratrol**  **Hemin**  **STA-4783**  **MG-132**  **Thapsigargin**  **Poly I:C** | 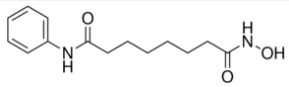  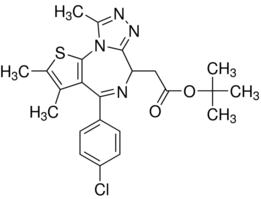  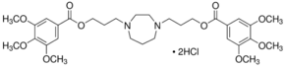  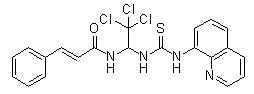  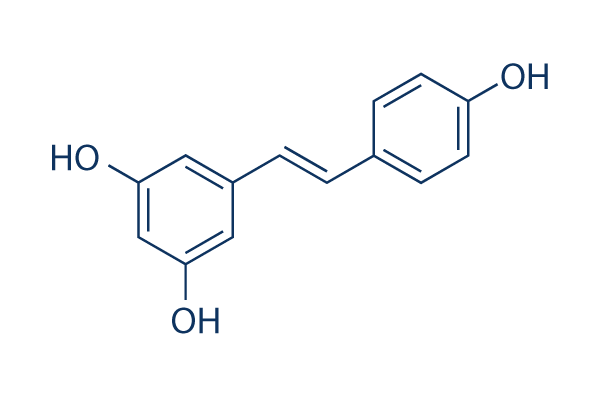  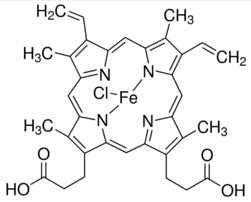  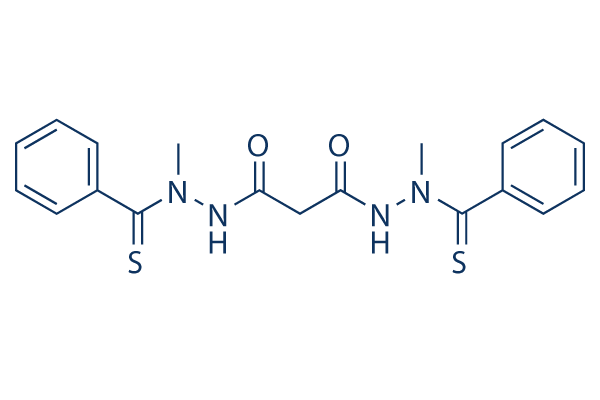  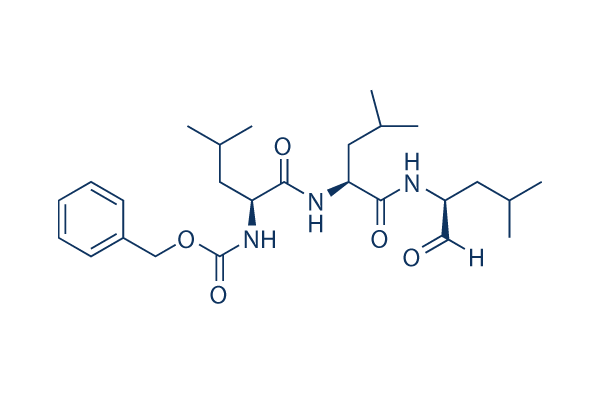  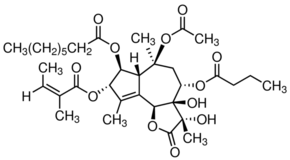 | HDAC  TLR/ NF-κB/NFAT  BRD4/p-TEFb  unknown  unknown  Sirt1  Proteasome  Unknown  Proteasome  Unknown  unknown |

**The pictures of compound structure were from suppliers’ official website.**

**Table S3. Characteristics of HIV-infected patients in this study**.

| **Donor no.** | **Sex** | **Age (y)** | **CD4 count (cells/μl)** | **Plasma viral load (copies/ml)** | **year of diagnostic** | **cART initiation and duration** | **Undetectable viral load** | **Treatments** |
| --- | --- | --- | --- | --- | --- | --- | --- | --- |
| 1 | M | 30 | 993 | < 20 | 2006 | since 2006 | 6y | tenofovir, lamivudine, nevirapine |
| 2 | F | 41 | 876 | < 20 | 2003 | since 2003 | 10y | zidovudine, lamivudine, efavirenz |
| 3 | M | 54 | 780 | < 20 | 2001 | since 2001 | 8y | zidovudine, lamivudine, lopinavir |
